# Development of a risk predictive score for intraoperative hypothermia in pediatric patients: A retrospective cohort study

**DOI:** 10.1371/journal.pone.0335796

**Published:** 2025-10-28

**Authors:** Worachet Saezhang, Maliwan Oofuvong, Nalinee Kovitwanawong, Kanlayanee Yongyukantorn, Nichanun Pichayanukul, Chareefar Lakateb

**Affiliations:** Department of Anesthesiology, Faculty of Medicine, Prince of Songkla University, Hat Yai, Songkhla, Thailand; Rutgers Robert Wood Johnson Medical School, UNITED STATES OF AMERICA

## Abstract

**Objective:**

This study aims to identify the risk factors and develop a risk predictive score of intraoperative hypothermia in pediatric surgery.

**Methods:**

This was a retrospective cohort study of children under the age of 12 years who underwent anesthesia in 2020 at a super-tertiary care hospital, Thailand. Those with one episode of body temperature 32−35°C or 35.1–35.9°C were defined as having mild and very mild hypothermia, respectively. Data, including patient demographics, clinical information, and perioperative data, were extracted from the hospital information system and were analyzed to identify potential risk factors of hypothermia. The variables associated with intraoperative hypothermia at a p-value <0.2 then were included in the multinomial logistic regression analysis between the two outcomes (mild and very mild hypothermia) (relative risk ratio [RRR] and 95% confidence interval [CI]). The predictors of mild hypothermia were included in the multivariate logistic regression analysis where the association of each risk factor was presented as an odd ratio (OR) and 95% CI.

**Results:**

Among the 940 eligible patients, 163 (17.34%) and 34 (3.62%) experienced intraoperative very mild and mild hypothermia, respectively. On multivariate analysis, intraoperative very mild hypothermia was associated with ASA physical status >3 (RRR: 6.4[2.9, 14.5]), anesthetic time >2 hours (RRR: 2.6[1.8, 3.8]), and major operation (RRR: 2.0[1.2, 3.4]) whereas intraoperative mild hypothermia was associated with ASA physical status >3 (adj OR: 8.01 [3.13, 20.5]), preoperative temperature >37.2°C (adj OR:3.3[1.5, 7.4]), anesthetic time >2 hours (adj OR:3.1[1.3, 7.4]), and no active warming (adj OR:9.3[2.9, 29.8]). A risk predictive score of mild hypothermia using a cut-point of 1.0 had a sensitivity and specificity of 85.9% and 52.53% respectively, with an area under the receiver operating characteristic curve of 0.78.

**Conclusions:**

Application of forced-warming after prolonged anesthesia, especially in high morbidity child, can reduce the risk of intraoperative hypothermia during pediatric surgery.

## Introduction

Inadvertent intraoperative hypothermia is a common problem, especially in pediatric anesthesia. Children and infants are prone to developing hypothermia intraoperatively [1]. Physiologic changes, such as the immature thermoregulatory capacity, plays a major role in poor temperature control. In addition, a high surface area causes more heat loss affected by the operating room environment for children compared to adults [2]. Activating sympathetic activity in response to hypothermia with increasing norepinephrine levels can lead to increased oxygen consumption [3]. There are few studies on pediatric perioperative hypothermia with limited risk factors and outcomes [4]. A study by Pearce et al. [5] found that invasive procedures, older age, longer duration of anesthesia, greater blood loss, and blood transfusion are associated with intraoperative hypothermia. Due to the dissimilarity of physiology in age groups, the age range of the patients in the previous study was very wide; the sample included infants and young adolescents.

The literature defines intraoperative hypothermia as a core temperature <36.0°C [6] or <35°C [7]. Until now, risk factors of pediatric hypothermia (temperature <36°C) are not well defined and there is currently no relevant risk predictive score available for use. Furthermore, a body temperature ≤35°C has been shown to be correlated with an increased risk of bradycardia, low platelet count, and coagulopathy compared to a cut-point of 36°C [8]. Therefore, we aimed to determine the risk factors of hypothermia in pediatric surgery using different cut-points (<36°C and **≤**35°C) and develop a risk predictive score based on the final model.

## Materials and methods

A single-center retrospective cohort study in children who underwent surgery in 2020 at super-tertiary care hospital in southern Thailand was performed after approval by the Institutional Ethics Committee of the Faculty of Medicine, Prince of Songkla University on 11 February 2022 (REC 65-003-8-4). Since this is a retrospective study, informed consent was waived by the Institutional Ethics Committee of the Faculty of Medicine, Prince of Songkla University. The data were accessed on 12 February 2022 after the Ethics Committee’s approval. All data were fully anonymized before being accessed by the investigators. The data, including patient demographics, anesthetic information, and perioperative data, was extracted from the hospital information system. The anesthetic records were collected and analyzed to identify the outcomes. The DOI link by Protocols.io is dx.doi.org/10.17504/protocols.io.q26g7mq71gwz/v1.

The inclusion criteria consisted of children aged less than 12 years who underwent any surgical procedure under anesthesia from 1 January to 31 December 2020 with an American Society of Anesthesiologists (ASA) physical status of 1–4. The exclusion criteria were as follows: (1) intraoperative hyperthermia (a rise in core temperature of 37.8°C); (2) children who underwent cardiopulmonary bypass with deliberate intraoperative hypothermia; (3) children with no temperature monitoring.

### Main outcome

Intraoperative temperature maintained at ≥36°C was considered as normothermia. Intraoperative temperature between 35.1–35.9 °C was considered as very mild hypothermia, while at least one episode of core temperature 32–35°C indicated mild hypothermia. The severity of intraoperative hypothermia was classified as mild hypothermia (32–35°C), moderate hypothermia (28–32°C), and severe hypothermia (20–28°C) [8]. Duration of hypothermia was defined as the period during which the core body temperature remained <36˚C measured in 30-minute intervals. If within any 30-minute period, the body temperature fluctuated from normal to below 36 ˚C, or vice versa, 15 minutes was added to the total duration of hypothermia. Temperatures were recorded in the anesthetic chart every 30 minutes by nurse anesthetists via nasopharyngeal, esophageal, or rectal probe (S1 Fig). For body temperatures measured in the tympanic membrane or axillary route, 0.5°C was added to correct the measurement close to core temperature [9]. For operation times exceeding 30 minutes, a forced-air warming system was used to prevent hypothermia in all patients.

### Explanatory and confounding factors

The following clinical variables that are known to be associated with intraoperative hypothermia were recorded: patient-related variables included gender, age, ASA physical status, body weight (kg), body height (cm), and body surface area (BSA) calculated using the Mosteller equation ($\sqrt{\frac{\text{Weight}(\text{kg}textrmxHeight(\text{cm})}{\text{3600}}}$) [10]. Weight-to-BSA ratio was defined as a ratio of weight (kg) to BSA. Baseline preoperative body temperature was defined as the last temperature recorded at the ward before the operation. Surgery-related variables included site of surgery, type of surgery (open and endoscopic), duration of surgery (the duration between surgical wound incision and surgical wound closure), and magnitude of surgery (major, intermediate, minor). Major surgery was defined as surgery in which body cavities or major vessels were exposed to an ambient temperature, for example major abdominal, thoracic, major vascular, and thoracic spine surgery with instrumentation. Intermediate surgery was defined as surgery in which body cavities were exposed to a lesser degree such as appendectomy and herniotomy. Minor surgery was defined as surgery involving superficial or endoscopic surgery [11]. Anesthesia-related variables included anesthetic agents used, anesthetic duration (the duration between an anesthetic drug given and extubation), estimated blood loss, blood transfusion, method of temperature monitoring (i.e., nasopharyngeal, esophageal, rectal, or skin sensors), and use of active forced-air warming.

### Statistical analysis

Data were entered in Epidata version 3.1 and analyzed in R version 4.2.1. All categorical variables were presented with frequency and percentage. Continuous variables were presented with mean and standard deviation. The chi-square test or Fisher’s exact test was used to compare categorical variables. Normally distributed data were compared with the unpaired Student’s t-test while the Wilcoxon rank-sum test was used to compare non-normally distributed data. The variables associated with intraoperative hypothermia at a p-value <0.2 were included in the initial multinomial logistic regression analysis to identify the dose-response relation between the two outcomes (mild and very mild hypothermia) using a backward stepwise selection method. The model with the lowest AIC was chosen as the final model. To improve model interpretability, continuous variables were dichotomized using the optimal cut-point from the receiver operating characteristic (ROC) curve. An ROC analysis was used to define an optimal cut-point using the highest Youden index. The predictors in the final model of mild hypothermia compared to normothermia and very mild hypothermia (as a reference) were included in the multivariate logistic regression analysis where the association of each risk factor was presented as an odd ratio and 95% confidence interval.

### Score derivation

A risk predictive scoring system was developed using the predictors derived from the final multivariate logistic regression model. The score of each predictor was derived from the coefficient and rounded to the nearest integer to obtain a maximum score of 10. In the final model, each predictor score was summarized for predicting intraoperative hypothermia. Youden’s index was used to evaluate the maximum specificity and sensitivity cut-off values of the predictive score. The performance of the final model was assessed using the area under the ROC curve (AUC). An AUC value of more than 80 indicated a very good performance of the model. The Hosmer-Lemeshow test was used to assess the goodness-of-fit and calibration of the final risk predictive model.

### Sample size calculation

We used the criteria as suggested by Riley et al. [12] to calculate the minimum sample size estimation required for developing a risk predictive model. Sample size estimation was based on the results from Hu ’s study [13] regarding the risk factors of intraoperative hyperthermia (defined as body temperature <36°C). We expected 10 predictors in our risk predictive model, therefore the minimum sample size required for new model development, based on the Hu ’s study, was 689 patients with 118 events by assuming an outcome prevalence was 0.18 and an event per predictor was 11.7 under an alpha level of 0.05 and a power of 80%.

## Results

**Fig 1:**
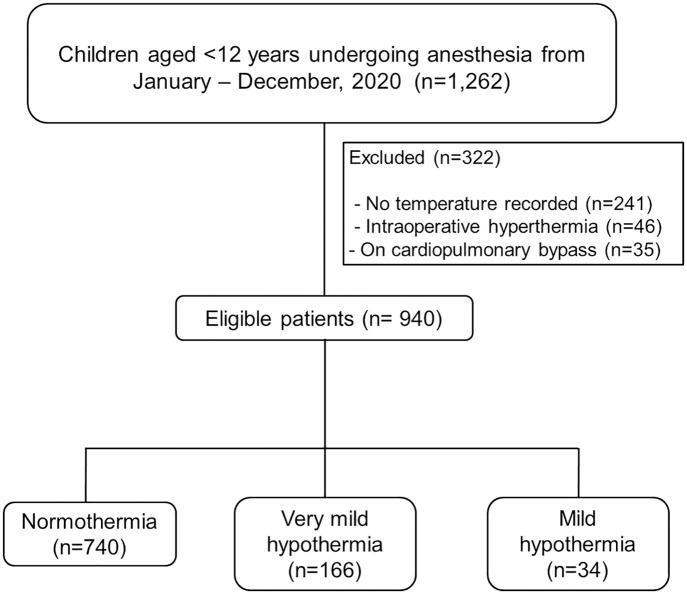
Flow diagram of the study.

From January to December 2020, 1,262 patients met the inclusion criteria, of which 322 were excluded. Among the 940 eligible patients, 166 (17.7%), and 34 (3.6%) experienced intraoperative very mild and mild hypothermia, respectively (Fig 1).

Table 1 shows the patient, anesthesia-, and surgery-related characteristics of the study sample. Age, body weight, height, and BSA in the very mild hypothermia group were significantly lower than those in the normothermia group. Most of the patients in the normothermia group had an ASA physical status 2 (50.9%) whereas patients in the mild hypothermia group had a higher ASA physical status (41.2% of ASA 3 and 38.2% of ASA 4). Baseline preoperative body temperature was a slightly higher in the mild hypothermia group compared to the other two groups (37.2 vs 36.8 °C, respectively). The proportion of patients receiving major surgeries in the normothermia group was significantly lower than those in the very mild and mild hypothermia groups (7.4%, 21.7%, 23.5%, respectively). Anesthetic time and operation time in the mild hypothermia group were significantly longer than those in the normothermia group. Intraoperative blood loss in the mild hypothermia group was significantly longer than those in the very mild hypothermia and the normothermia group (35, 5, and 5 mL, respectively). Active warmer use in the normothermia and very mild hypothermia group was significantly higher than those in the mild hypothermia group (98%, 98%, 74%, respectively).

**Table 1:** Patient baseline characteristic, anesthesia-, and surgery-related predictors (N = 940).

Characteristic	Normothermia (N = 740)	Very mild hypothermia (N = 166)	Mild hypothermia (N = 34)	p-value
Male	480 (64.9%)^c^	111 (66.9%)^a^	15 (44.1%)^a,c^	0.037*
Age (year)	4.4 [1.8, 7.7]^b^	3.7 [0.83, 7.7]^b^	3.9 [0.40, 10.3]	0.10
Body weight (kg)	15.5 [10.0, 22.6]^b^	13.1 [8.0, 21.7]^b^	16.6 [5.05, 25.0]	0.019**
Body height (cm)	102.0 [80.0, 122.0]^b^	94.0 [70.0, 120.0]^b^	101.5 [58.1, 145.0]	0.035**
BSA (m^2^)	0.7 [0.5, 0.9]^b^	0.6 [0.4, 0.8]^b^	0.7 [0.3, 1.0]	0.015**
Weight to BSA ratio (kg/ m^2^)	23.4 [21.0, 26.5]^b^	22.9 [19.3, 25.6]^b^	24.1 [16.6, 25.0]	0.018**
ASA physical status				<0.001***
1	158 (21.4%)	34 (20.5%)	5 (14.7%)	
2	377 (50.9%)	65 (39.2%)	2 (5.9%)	
3	188 (25.3%)	49 (30.1%)	14 (41.2%)	
4	17 (2.3%)^a,b,c^	18 (10.8%) ^a,b,c^	13 (38.2%) ^a,b,c^	
Preoperative BT (°C)	36.8 [36.6, 37.0]^c^	36.8 [36.6, 37.0]^a^	37.2 [36.6, 37.9]^a,c^	0.010**
Site of surgery				<0.001***
Superficial	120 (16.2%)	18 (11.0%)	4 (11.8%)	
Eye, ear-nose-throat	318 (43.0%)	47 (28.3%)	4 (11.8%)	
Abdomen	127 (17.2%)	36 (21.7%)	4 (11.8%)	
Extremities	136 (18.4%)	40 (24.1%)	13 (38.2%)	
Intracranial	18 (2.4%)	9 (5.4%)	5 (14.7%)	
Intrathoracic	22 (2.8%)	16 (9.6%)	4 (11.8%)	
Magnitude of Surgery				<0.001***
Major	55 (7.4%)^b,c^	36 (21.7%)^b^	8 (23.5%)^c^	
Intermediate	521 (70.4%)	113 (68.1%)	25 (73.5%)	
Minor	164 (22.2%)	17 (10.2%)	1 (2.9%)	
Emergency surgery	108 (14.6%)^c^	30 (18.1%)	11 (32.4%)^c^	0.015*
GA with				0.5
ETT intubation	639 (86.4%)	139 (83.7%)	31 (91.2%)	
LMA	101 (13.6%)	27 (16.3%)	3 (8.8%)	
TIVA use	26 (3.5%)	1 (0.6%)^b^	2 (5.9%)^b^	0.042
Anesthetic time (min)	105.0 [80.0, 146.2]^a,b,c^	140.0 [96.3, 185.0] ^a,b,c^	157.5 [120.0, 193.8] ^a,b,c^	<0.001**
Operation time (min)	70.0 [50.0, 110.0] ^a,b,c^	100.0 [60.0, 135.0] ^a,b,c^	115.0 [86.3, 140.0] ^a,b,c^	<0.001**
Crystalloid (mL)	170.0 [100.0, 300.0]	150.0 [60.3, 307.5]	205.0 [73.0, 492.5]	0.6
Colloid	7 (0.9%)	4 (2.5%)	0 (0%)	0.2
Blood loss (mL)	5.0 [1.00, 15.0] ^a,b,c^	5.0 [2.00, 20.0] ^a,b,c^	35.0 [5.00, 137.5] ^a,b,c^	< 0.001**
Active warming	725 (98.0%)^c^	163 (98.2%)^a^	25 (73.5%)^a,c^	< 0.001*
Route of BT monitoring				0.01***
Rectal	72 (9.7%)	15 (9.1%)	3 (9.1%)	
Nasopharynx	163 (22.1%)	42 (25.5%)	9 (27.3%)	
Esophagus	257 (34.8%)	56 (33.9%)	15 (45.5%)	
Skin/axillary/tympanic membrane	246 (33.3%)	52 (31.5%)	6 (18.2%)	
Unknown	2	1	1	

**Note**: Data are presented as frequency (%) or median [interquartile range] unless stated otherwise. * < 0.05 by Chi-square test, ** < 0.05 by Wilcoxon ranksum test, *** < 0.05 by Fisher’s exact test. *IQR* Interquartile range, *ASA* American Society of Anesthesiologists*, BSA* Body surface area, *BT* Body temperature, *GA* General anesthesia, *ETT* endotracheal tube, *LMA* Laryngeal mask airway, *TIVA* Total intravenous anesthetic agent. a = significant difference between very mild hypothermia and mild hypothermia, b = significant difference between very mild hypothermia and normothermia, c = significant difference between mild hypothermia and normothermia

Table 2 shows the characteristic of the hypothermia group. Patients in the mild hypothermia group had a longer duration of hypothermia than those in the very mild hypothermia group (82.5 vs 55 min).

**Table 2:** The characteristic of hypothermia group (N = 200).

Characteristic	Very mild hypothermia (N = 166)	Mild hypothermia (N = 34)
Nadir temperature (°C)	35.6 [35.5, 35.8]	34.9 [34.5, 35.0]
Duration of hypothermia (min)	55.0 [30.0, 67.5]	82.5 [60.0, 115.0]
Severity of hypothermia		
Mild	166 (100%)	34 (100%)

**Note**: Data are presented as frequency (%) or median [interquartile range] unless stated otherwise. *IQR* Interquartile range.

A comparison of factors associated with intraoperative hypothermia are shown in Table 3. Patient-related factors (male, weight to BSA ratio, and baseline preoperative body temperature), surgery-related factors (emergency surgery, magnitude of surgery, site of the operation, operation time), and anesthetic-related factors (ASA physical status, total intravenous anesthetic agent use, anesthetic time, blood loss, and use of active warmer) with a p-value less than 0.2 were included in the initial multivariate model. Continuous variables were dichotomized, i.e., preoperative body temperature (>37.2 vs ≤ 37.2°C), weight to BSA ratio (<16 vs ≥ 16 kg/m^2^), and anesthetic time (< 120 vs ≥ 120 min).

**Table 3:** Multinomial logistic regression analysis of risk factors associated with intraoperative hypothermia (N = 940).

Predictors	Very mild hypothermia (Ref: Normothermia)		Mild hypothermia (Ref: Normothermia)		LR p-value
Beta coefficient	RRR (95%CI)	Beta coefficient	RRR (95%CI)	
ASA physical status >3	1.86	6.42 (2.85, 14.5)***	2.79	16.2 (5.7, 46.2)***	<0.001
Preoperative BT > 37.2°C	−0.59	0.56 (0.31, 1.0)*	0.97	2.63 (1.15, 6.05) *	0.05
Major operation	0.7	2.01 (1.21, 3.35)**	0.61	1.84 (0.69, 4.93)	0.025
Anesthetic time > 120 min	0.96	2.62 (1.81, 3.79)***	1.4	4.05 (1.7, 9.65)**	<0.001
TIVA	−2.31	0.1 (0.01, 0.83)*	−0.06	0.94 (0.16, 5.56)	0.01
No active warming	0.1	1.1 (0.29, 4.16)	2.32	10.1 (2.97, 34.7)***	0.002

**Note**: *** p-value <0.001 by Wald test, ** represent p-value <0.01 by Wald test,* represent p-value <0.05 by Wald test. *Ref* Reference*, RRR* Relative risk ratio, *CI* Confidence interval, *LR* Likelihood ratio, *ASA* American Society of Anesthesiologists*, BT* Body temperature, *TIVA* Total intravenous anesthetic agent.

### Multivariate logistic regression analysis of risk factors associated with intraoperative mild hypothermia

The predictors in the final multinomial logistic model of mild hypothermia compared to normothermia and very mild hypothermia (baseline group) were included in the multivariate logistic regression model for mild hypothermia outcome (S1 Table). Mild hypothermia was associated with ASA physical status >3, preoperative body temperature >37.2°C, anesthetic time >120 min and no active warming use. The final model showed a very good performance with an AUC of 0.84. (S2 Fig)

### Development of a risk predictive score for intraoperative mild hypothermia

**Fig 2:**
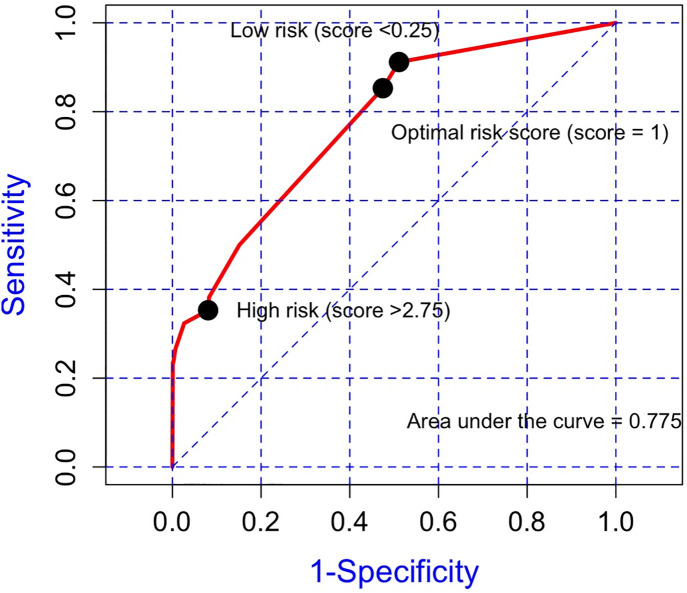
Receiver operating characteristic curve based on the risk predictive score for predicting intraoperative hypothermia, optimal cut-off points, and area under the curve.

Significant predictors derived from the multivariate logistic analysis model were included in the regression coefficient-based model which are shown in S1 Table (Table 4). The risk predictive score values ranged from 0–6. The final risk predictive model showed a good discriminative ability to distinguish patients with intraoperative mild hypothermia, with an AUC of 0.78. A cut-off point of 1 was chosen, which was closest to the top-left part of the ROC analysis. The sensitivity, specificity, accuracy, positive predictive value, and negative predictive value of the predictive score model were 85.3, 52.5, 80.6, 11.7, and 98.5%, respectively. (Fig 2)

**Table 4:** Risk predictive score using the regression coefficient-based model of mild hypothermia (N = 940).

Predictors	Regression coefficient	Score
ASA physical status >3	2.0806	3
Preoperative BT > 37.2°C	1.1965	1.5
Anesthetic time > 120 min	1.1445	1.5
Major operation	0.3374	0.5
TIVA	0.5286	0.5
No active warming	2.2246	3

**Note:** *ASA* American Society of Anesthesiologists*, BT* Body temperature, *TIVA* Total intravenous anesthetic agent.

The degree of risk of intraoperative mild hypothermia was classified into high-risk patients who had a risk score of >2.75 (sensitivity 35.3% and specificity 92.0%), intermediate-risk patients who had a risk score of 0.25–2.75, and low-risk patients who had a risk score of <0.25 (sensitivity 91.2% and specificity 48.9%). The chi-square value of the Hosmer-Lemeshow test was 1.5742 (p = 0.455), corresponding to good model calibration.

## Discussion

The incidence of intraoperative hypothermia in pediatric surgery in our study was 21.3% (200/940). The incidence of very mild (core body temperature 35.1–35.9°C) and mild hypothermia (core body temperature 32–35°C) was 17.7% and 3.6%, respectively. Despite the controversial definition of intraoperative hypothermia, the awareness of the adverse consequences of intraoperative hypothermia has been increasing. Although the cut-off point of 36°C is broadly accepted in the literature, we performed a risk predictive score using the cut-point of ≤35°C for the following reasons. First, a recent meta-analysis in adults found that the cut-point of 36°C was not an adequately sensitive criterion for intraoperative hypothermia and overestimated the detrimental outcome of intraoperative hypothermia [14,15]. Second, Bindu et al. reported more significant outcomes in terms of bradycardia and coagulopathy compared to higher values for the cut-point [8]. Third, according to our multivariate analysis (Table 3), the predictors of very mild and mild hypothermia in children were similar; mild hypothermia was associated with higher risk of each predictor compared to the lower severity of hypothermia (very mild). In this study, only children aged <12 years were included since the skin surface area of children aged ≥12 years is considered to be similar to adults [16] and we wanted a more homogeneous sample of pediatric patients in our analysis.

All of the patients in our study experienced only mild or very mild hypothermia because the forced-air warming system is routinely used in pediatric surgery (98% in very mild hypothermia and 73.5% in mild hypothermia).

Due to the dissimilarity of physiology in age groups, risk factors of intraoperative hypothermia in adult studies may not be applicable to pediatric patients. However, there are only a few studies that explored the risk factors in the pediatric population [5,17]. Our study was the first to date to perform the risk predictive scores for predicting intraoperative hypothermia in pediatric patients.

### ASA Physical status

ASA physical status was used to categorize the patient based on the severity of co-existing diseases. Wongyingsinn et al. [18] demonstrated that adult patients with ASA physical status 3–4 had a higher incidence of intraoperative hypothermia. This result was similar to other studies in adults [19,20]. Zhao et al. [17] also found an association between higher ASA physical status and intraoperative hypothermia in neonates and infants. Patients with a higher ASA physical status had poor thermoregulatory control and were prone to develop vasodilatation from co-existing medical diseases resulting in intraoperative hypothermia.

Pediatric patients who had an ASA physical status >3 would have a risk score of 3, which is classified as high-risk. Temperature monitoring should be rigorously considered along with preoperative active warming application, and intraoperative warming protocols including active forced-air warming and use of intravenous fluid warming devices.

### Preoperative baseline temperature

Preoperative baseline temperature >37.2°C was found to be associated with intraoperative hypothermia. In contrast, previous studies showed the opposite result [21]. Yi et al. [21] showed that higher baseline core temperature before induction was a protective factor for intraoperative hypothermia among adults; however, infectious causes of preoperative hyperthermia were excluded in their study. Nonetheless, a study by Cho et al. [22], also in adults, found that lower baseline core temperature before anesthesia was a protective factor of intraoperative hypothermia in orthopedic surgery. The median baseline temperature in Cho’s study was 36.8°C [36.6, 36.9°C], which was lower than the baseline temperature of children in our study (37.2°C [36.6, 37.7°C]). Although we excluded intraoperative hyperthermia patients (core temperature ≥37.8°C), 30 minutes of temperature recording may not be sensitive enough for retrospective data if the rise of temperature occurrence was in between. Hyperthermia is the thermoregulatory response to systemic inflammation [23]. During the perioperative period, vasodilation from systemic inflammatory response may be exaggerated, and thermoregulatory capacity was also suppressed by various anesthetic agents leading to sub-temperature.

Pediatric patients who had a preoperative body temperature ≥37.2°C would have a risk score of 1.5 indicating an intermediate-risk patient. Methods to decrease the preoperative temperature, such as application of a tepid sponge, prescribing oral paracetamol, or postponing elective surgery, may reduce a patient’s risk of intraoperative hypothermia.

### Anesthetic time

Our results showed that the duration of anesthesia was independently associated with intraoperative hypothermia, a result supported by other studies. [24,25]. In addition, Zhao et al. [17] reported that operation times longer than 60 minutes increased the odds of intraoperative hypothermia by two-fold. Prolonged duration of anesthesia may lead to longer exposure to ambient operating room temperatures and may necessitate the administration of additional unwarm intravenous fluid.

Pediatric patients who had undergone a long anesthetic duration would have a risk score of 1.5 indicating an intermediate-risk patient. A preoperative active warming protocol might be warranted in these cases. Additionally, intraoperative hypothermia prevention methods, including warming intravenous fluid, active forced-air warming use, warm operating room temperature, and warming fluid irrigation, may reduce the risk of intraoperative hypothermia.

### Active forced-air warming

In various studies in adults, active forced-air warming was considered as a strong protective factor against intraoperative hypothermia [26–28]. Using an active convection warming method, active forced-air warming transmits heat by blowing warm air through a blanket, aiming to prevent heat loss during the operation. Lee et al. [29] reported that the use of forced-air warming reduced the incidence of intraoperative hypothermia in pediatric patients (age < 16 years). In addition, Su et al. [30] found that forced-air warming was more effective than passive insulation in preventing adverse consequences of intraoperative hypothermia. Corresponding to previous studies, our data showed that if no active forced-air warming was used, the risk of intraoperative hypothermia increased more than nine-fold.

Non-use of an active forced-air warming system indicates a high risk (score of 3) of developing intraoperative hypothermia in pediatric patients. An active forced-air warming system should be used in all children for both preoperative and intraoperative periods to reduce the risk of adverse consequences.

### Implications of the study

This risk predictive score developed in our study is feasible to use and could be easily adopted in the perioperative period. Observations of high-risk patients using our model may help in the early detection of intraoperative hypothermia and prevent further adverse consequences. In resource-limited countries, additional interventions may not be possible in all cases. Since a risk score of >1 increased the risk of intraoperative hypothermia in pediatric patients, only one predictor such as ASA physical status >3, anesthetic time >2 hours, preoperative body temperature >37.2°C, or absence of active warming methods may lead to intraoperative hypothermia. Patients with a risk score of more than 1 should have their temperature monitored rigorously to maintain normothermia, especially high-risk patients who have a risk score of >2.75.

Active forced-air warming and high preoperative body temperature >37.2°C are modifiable. In our study, early warming interventions by an active forced-air warming decreased the risk of intraoperative hypothermia. An attempt to decrease preoperative body temperature such as use of a tepid sponge, prescribing oral paracetamol, and postponing an elective operation may reduce the risk of developing intraoperative hypothermia.

### Strengths and limitations

As this was a retrospective cohort study, some data such as the precise ambient temperature of an operating room (approximately 22–24°C), the amount of fluid irrigation, the temperature of the fluid, and the waiting time before the operation could not be collected. Skin temperature (skin, axillary, or tympanic membrane), which serves as one-third of the temperature measurement, could make less accuracy to the temperature measurement compared to the core temperature measurement. However, we adjusted skin temperature readings by adding 0.5°C, based on previous literature (axillary +0.55°C [9]; tympanic membrane +0.42°C [31]). This adjustment may help better approximate core temperature in the limitation of retrospective study. In addition, body temperature was recorded in 30 minute-intervals, which may be too long to observe rapid temperature fluctuations. Due to the small number of patients in the mild hypothermia group (34 cases), the results of the multivariate model may be underpowered. Implications of this study should be used in light of these limitations. Finally, due to the single center study, the external validation of the prediction scoring system might be limited. Additionally, further prospective cohort studies for validation data would be conducted to validate our risk predictive score for clinical practice.

## Conclusions

Mild hypothermia was associated with a higher risk compared to the lower severity of hypothermia. Risk factors associated with intraoperative hypothermia were ASA physical status >3, anesthetic time >120 minutes, preoperative body temperature >37.2°C, and the absence of active warming, the last two factors being modifiable. A risk predictive score with a cut-point of 1.0 can help physicians in the early detection of intraoperative hypothermia and reduce the risk of adverse outcomes.

## Supporting information

S1 Fig Anesthetic record with temperature monitoring. (TIF)

S1 Table Multivariate logistic regression analysis of risk factors associated with mild hypothermia compared to normal and very mild hypothermia (N = 940). (DOCX)

S2 Fig Receiver operating characteristic curves based on the predictors associated with intraoperative mild hypothermia and respective areas under the curves. (JPG)

S1 File Hypothermia minimal data set. (CSV)
