# Supplementary figures and images for: Development of a risk predictive score for intraoperative hypothermia in pediatric patients: A retrospective cohort study
Source: PLoS One. 2025 Oct 28;20(10):e0335796. doi: 10.1371/journal.pone.0335796 (PMC12561954; doi:10.1371/journal.pone.0335796)

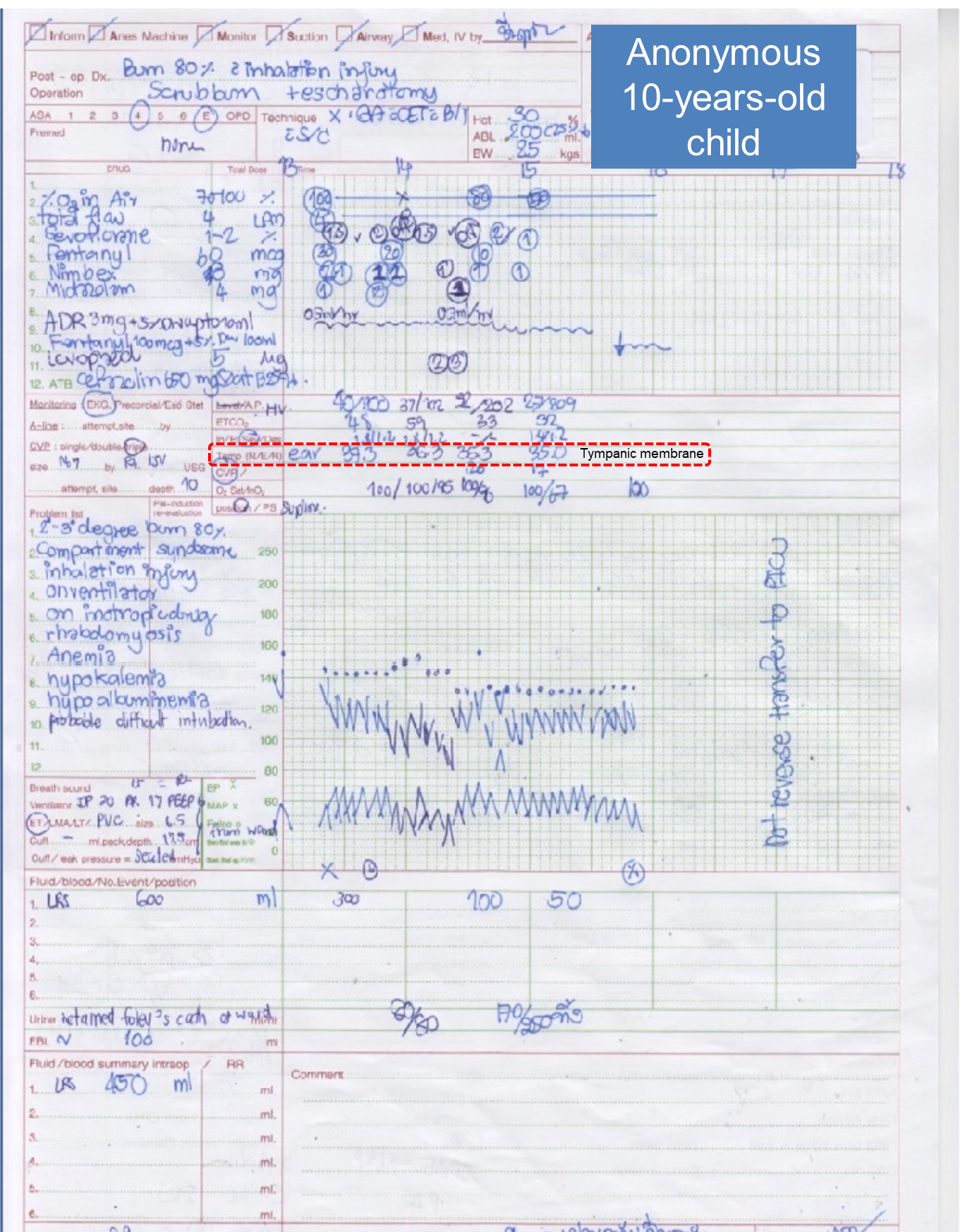

Supplement: S1 Fig — (TIF) [file pone.0335796.s001.tif]

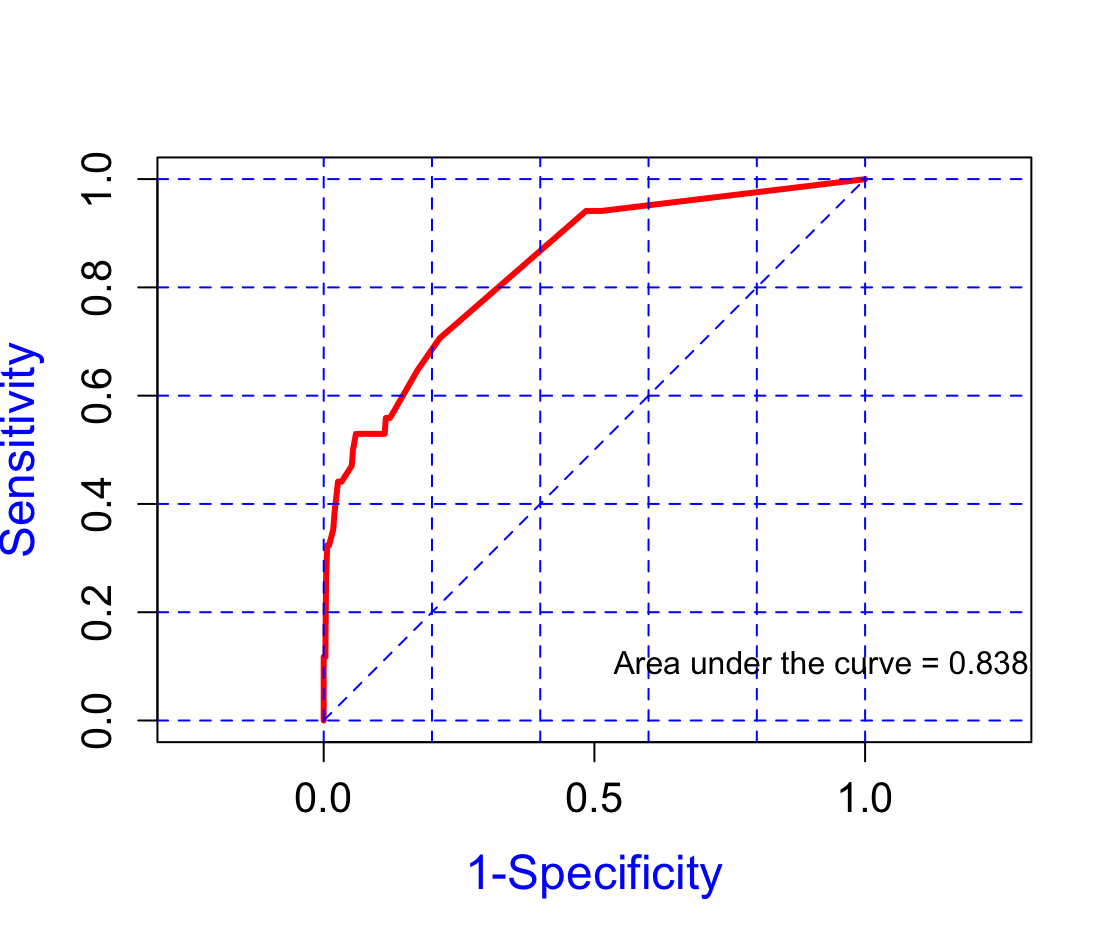

Supplement: S2 Fig — (JPG) [file pone.0335796.s003.jpg]
