# Supplementary material for: Development of a risk predictive score for intraoperative hypothermia in pediatric patients: A retrospective cohort study
Source: PLoS One. 2025 Oct 28;20(10):e0335796. doi: 10.1371/journal.pone.0335796 (PMC12561954; doi:10.1371/journal.pone.0335796)
Supplement: S1 Table — (DOCX) [file pone.0335796.s002.docx]

**Supplemental file 2.** Multivariate logistic regression analysis of risk factors associated with mild hypothermia compared to normal and very mild hypothermia (N=940)

| Predictors | Crude OR (95%CI) | Adjusted OR (95%CI) | LR p-value |
| --- | --- | --- | --- |
| ASA physical status >3  (Ref: ≤3) | 15.4 (7.13, 33.3) | 8.01 (3.13, 20.5) | < 0.001 |
| Preoperative BT > 37.2°C  (Ref: ≤37.2°C) | 6.57 (3.25, 13.3) | 3.31 (1.47, 7.44) | 0.006 |
| Anesthetic time >120 min  (Ref: ≤ 120 min) | 3.54 (1.67, 7.49) | 3.14 (1.34, 7.37) | 0.006 |
| Major operation | 2.76 (1.21, 6.27) | 1.4 (0.53, 3.73) | 0.60 |
| TIVA | 2.03 (0.46, 8.93) | 1.7 (0.32, 8.98) | 0.552 |
| No active warming | 17.8 (7.27, 43.4) | 9.25 (2.87, 29.8) | <0.001 |

**Note:** *Ref* Reference*, OR* Odd ratio, *CI* Confidence interval, *LR* Likelihood ratio, *ASA* American Society of Anesthesiologists*, BT* Body temperature, *TIVA* Total intravenous anesthetic agent.
